# Detection of Senecionine in Dietary Sources by Single-Use Electrochemical Sensor

**DOI:** 10.3390/mi12121585

**Published:** 2021-12-20

**Authors:** Huseyin Senturk, Ece Eksin, Ulvi Zeybek, Arzum Erdem

**Affiliations:** 1 Department of Analytical Chemistry, Faculty of Pharmacy, Ege University, Izmir 35100, Turkey; mhuseyinsenturk@hotmail.com (H.S.); ece.eksin@idu.edu.tr (E.E.); 2 Biomedical Device Technology Programme, Vocational School of Health Sciences, Izmir Democracy University, Izmir 35140, Turkey; 3 Department of Pharmaceutical Botany, Faculty of Pharmacy, Ege University, Izmir 35100, Turkey; ulvi.zeybek@ege.edu.tr

**Keywords:** senecionine, pyrrolizidine alkaloids, electrochemical sensor, differential pulse voltammetry, natural toxins

## Abstract

Pyrrolizidine alkaloids (PAs) are produced by plants as secondary compounds that are the most widely distributed natural toxins. There have been many cases of human toxicity caused by consumption of toxic plant species, as herbal teas and grain or grain products contaminated with PA-containing seeds have been reported. Companies that produce dried spices and tea leaves should examine the PA level in their products. For the first time in the literature, a simple and inexpensive electrochemical assay based on a single-use sensor was introduced for quantitative determination of senecionine (SEN) in the most frequently contaminated food sources. SEN was immobilized on a pencil graphite electrode surface by the passive adsorption technique. Differential pulse voltammetry (DPV) was used to evaluate the oxidation signal of SEN, which was observed to be around +0.95 V. The oxidation signal was specific to the SEN in the sample, and the current value was proportional to its concentration. The selectivity of our assay was also tested in the presence of other similar PAs such as intermedine, lycopsamine, and heliotrine. The detection limit is calculated by developed assay and found to be 5.45 µg/mL, which is an acceptable concentration value of SEN occurring at toxic levels for consumers. As an application of the developed sensor in food products, the electrochemical detection of SEN was successfully performed in flour and herbal tea products.

## 1. Introduction

Pyrrolizidine alkaloids are the secondary metabolites produced by plants for defense against other organisms. These alkaloids are found in Asteraceae, Boraginaceae, and Fabaceae families and have a wide distribution in the world [1,2]. Pyrrolizidine alkaloids have toxic effects and can cause many negative effects on living things. The most common pyrrolizidine alkaloids are found these species *Tussilago farfara*, *Senecio* sp., *Petasites* sp., *Eupatorium* sp., *Heliotropium* sp., *Borago officinalis*, *Echium* sp., and *Symphytum* sp. [3]. Due to the toxic effects of pyrrolizidine alkaloids, various limitations have been introduced for these alkaloids. According to the recommendation of the E Commission [4], the daily oral intake level of pyrrolizidine alkaloids (containing 1, 2 unsaturated forms) and herbal preparations of N-oxide forms (teas and infusions) should not exceed 10 µg. In addition, 1 µg should not be exceeded in herbal extracts. Additionally, pyrrolizidine alkaloids that have retronecine (senecionine) and heliotridine bases are the alkaloids that require the most attention according to EFSA reports [5].

Since the structures of pyrrolizidine alkaloids are very similar to each other, their mechanisms, toxic effects, and binding to DNA occur in a similar way. The different toxicities of these alkaloids change the severity of the toxic effect on the body [6,7]. Pyrrolizidine alkaloids have hepatotoxic, carcinogenic, and genotoxic effects. Numerous articles have been published in the literature showing these effects [8,9,10]. Many cases of poisoning with pyrrolizidine alkaloids have been reported worldwide [11,12,13,14]. In addition, many cases of poisoning of animals such as cattle, sheep, and horses with pyrrolizidine alkaloids have been reported [15,16].

Pyrrolizidine alkaloids can be found in foods such as tea, flour, milk, honey, and meat. Therefore, the detection of pyrrolizidine alkaloids in foods is extremely important [17]. Senecionine is also a pyrrolizidine alkaloid and is one of the highly toxic pyrrolizidine alkaloids. Although senecionine is mainly found in *Jacobaea vulgaris* (*Senecio jacobaea*), it is also isolated from *Brachyglottis repanda*, *Erechtites hieraciifolius*, *Petasites*, *Syneilesis*, *Crotalaria*, and *Caltha leptosepala* species [18].

There is no report on the electrochemical detection of Senecionine and other pyrrolizidine alkaloids in the literature. Apart from this, there are studies developed by traditional detection methods such as chromatography, NMR, and mass spectroscopy, etc. [18,19,20].

For the first time in the literature, an electrochemical assay was developed in the present study for voltammetric determination of senecionine (SEN) based on the changes in the oxidation signal of SEN. The effect of a solution of DMSO and methanol upon the response of an electrochemical sensor and the immobilization time of SEN onto the surface of a single-use pencil graphite electrode (PGE) was investigated, while measuring the signal by using differential the pulse voltammetry (DPV) technique. The signal magnitude was proportional to the concentration that was used for quantitative analysis of SEN. Under the optimum conditions, the limit of detection (LOD) of SEN in buffer medium was calculated. The selectivity of our assay was tested in the presence of intermedine, lycopsamine, and heliotrine, which are similar PAs to SEN. The application of our electrochemical sensor to senecionine determination was also performed successfully in samples of herbal tea and flour, and the detection limit of SEN was calculated.

## 2. Materials and Methods

### 2.1. Apparatus

Electrochemical measurements were performed with µAUTOLABIII in combination with NOVA 1.11.1 software. All measurements were performed in a Faraday cage (Eco Chemie, Utrecht, The Netherlands). In the three-electrode system, PGE was used as working electrode, Ag/AgCl (BAS, Model RE-5B, West Lafayette, LA, USA) as reference electrode, and platinum wire as counter electrode.

### 2.2. Chemicals

Senecionine (SEN, PhytoLab GmbH & Co. KG, Vestenbergsgreuth, Germany) and standard solutions of intermedine, lycopsamine, and heliotrine were kindly donated from SIA Analysis Laboratories (Izmir, Turkey). The stock solutions of SEN (1 mg/mL) were prepared by dissolving SEN in dimethyl sulfoxide (DMSO) or methanol. The stock solutions of other PAs—intermedine and lycopsamine—were dissolved in methanol, and heliotrine was dissolved in DMSO. All the stock solutions of PAs were stored at +4 °C in dark place for the required period in our study.

### 2.3. Procedure

An electrochemical pretreatment step was first performed by applying +1.40 V during 30 s in 0.5 M acetate buffer solution (ABS, pH 4.80) in order to activate carboxylic acid groups of PGE, as reported in our previous studies [21,22]. Then, the electrodes were dipped into vials containing 40 µL SEN solution in order to immobilize SEN onto the electrode surface during 30 min by passive adsorption. Immobilization probably occurred because of hydrogen bonding between the hydroxyl group on the SEN and the carboxyl group on the PGE. Then, the electrodes were washed with PBS for 5 s to remove non-specific adsorptions. Differential pulse voltammetry (DPV) was used to evaluate the oxidation signal of SEN, which was observed to be around +0.95 V in PBS (0.05 M, pH 7.40). This oxidation signal was specific to the SEN in the sample, and the signal magnitude was proportional to the concentration that was used for quantitative analysis of SEN.

### 2.4. Sample Preparation

The products of flour (wheat) and herbal tea (i.e., linden tea) were purchased from a local supermarket in Turkey. After the preparation of each sample by following the proper procedure as mentioned below, the standard addition method was applied by addition of the required amount of SEN standard solution into the sample.

#### 2.4.1. Flour

The 0.5% (*v*/*w*) solution of flour was prepared in PBS (0.05 M, pH 7.40) and mixed for 5 min, as reported in our previous work [23].

#### 2.4.2. Aqueous Extraction of Herbal Tea Sample

An aqueous extraction of linden tea was carried out as reported in earlier works [24,25]. A portion of 200 mg of tea sample was weighed and transferred to a beaker containing 50 mL of ultrapure water and kept in a water bath at 80 °C with constant stirring for about 30 min, then filtered off in order to obtain a clear tea extract. The tea extract was used in electrochemical sensing after appropriate dilution (i.e. 0.5%) with PBS (0.05 M, pH 7.40).

### 2.5. Electrochemical Measurement

Measurement of the electrochemical signal of SEN was performed by the DPV technique in combination with PGE in PBS (0.05 M, pH 7.40) by scanning from +0.30 V to +1.40 V at the pulse amplitude of 50 mV with a scan rate of 50 mV/s.

Cyclic voltammetry (CV) measurements were performed by scanning from −0.80 V to +1.30 V, and a scan rate of 50 mV/s in 10 µg/mL SEN solution prepared in PBS (0.05 M, pH 7.40).

## 3. Results and Discussion

First, the electrochemical response of SEN was investigated by cyclic voltammetry (Figure S1). In the absence of SEN, there was no anodic peak in the working potential range, as expected (Figure S1a). However, in presence of 10 µg/mL SEN, there was an anodic peak observed at around +1.0 V vs. Ag/AgCl. The average signal was measured as 2.36 ± 0.02 µA (RSD%, 1%, *n* = 2), which belonged to the oxidation of SEN (Figure S1b).

**Figure 1:**
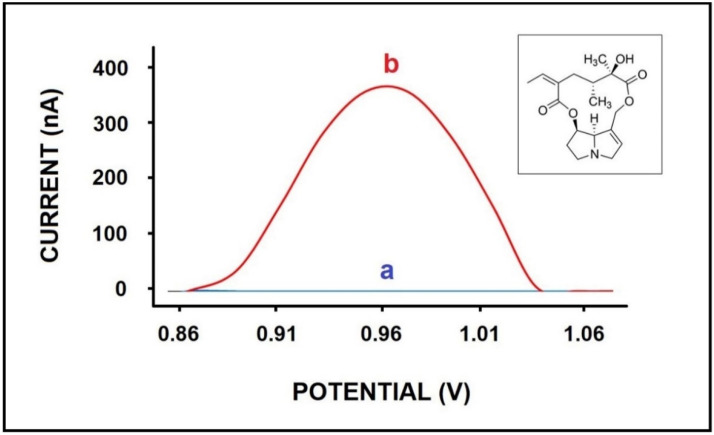
Voltammograms of (a) control experiment performed by PGE in PBS in the absence of SEN; (b) oxidation signal of 50 µg/mL SEN. Inset figure presenting the chemical structure of SEN.

The oxidation signal of 50 µg/mL SEN was measured at the peak potential of +0.97 V (Figure 1(b)) and measured as 233.33 ± 19.52 nA (RSD%, 8%, *n* = 6) using the differential pulse voltammetry (DPV) technique. The control experiments were performed in the absence of SEN, and no oxidation signal was observed. (Figure 1(a)).

**Figure 2:**
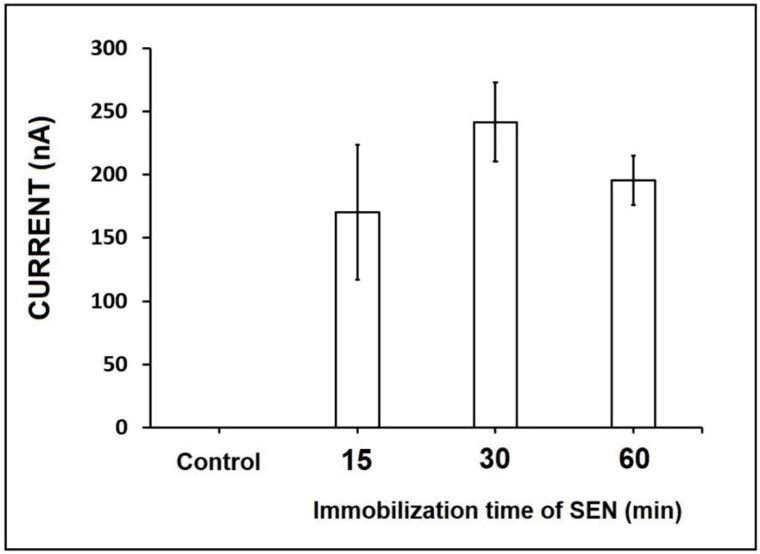
Histograms representing the result in the absence of SEN (control experiment performed in PBS alone) and the average oxidation signal of 50 µg/mL SEN (*n* = 3) after its immobilization onto the PGE surface for 15 min, 30 min, and 60 min immobilization time.

The effect of immobilization time on the immobilization efficiency of SEN onto the PGE surface was examined based on changes in the response. An amount of 50 µg/mL SEN (its stock solution was prepared in DMSO) was immobilized onto the electrode surface during 15, 30, and 60 min, and the oxidation signal of SEN was measured respectively as 200.50 ± 74.39 nA (RSD%, 37%, *n* = 3), 241.67 ± 31.07 nA (RSD%, 12%, *n* = 3), and 197.80 ± 40.08 nA (RSD%, 20%, *n* = 3) (Figure 2 and Figure S2). Since the highest and reproducible oxidation signal of SEN was measured in 30 min immobilization time (Figure 2), it was chosen as the optimum immobilization time.

**Figure 3:**
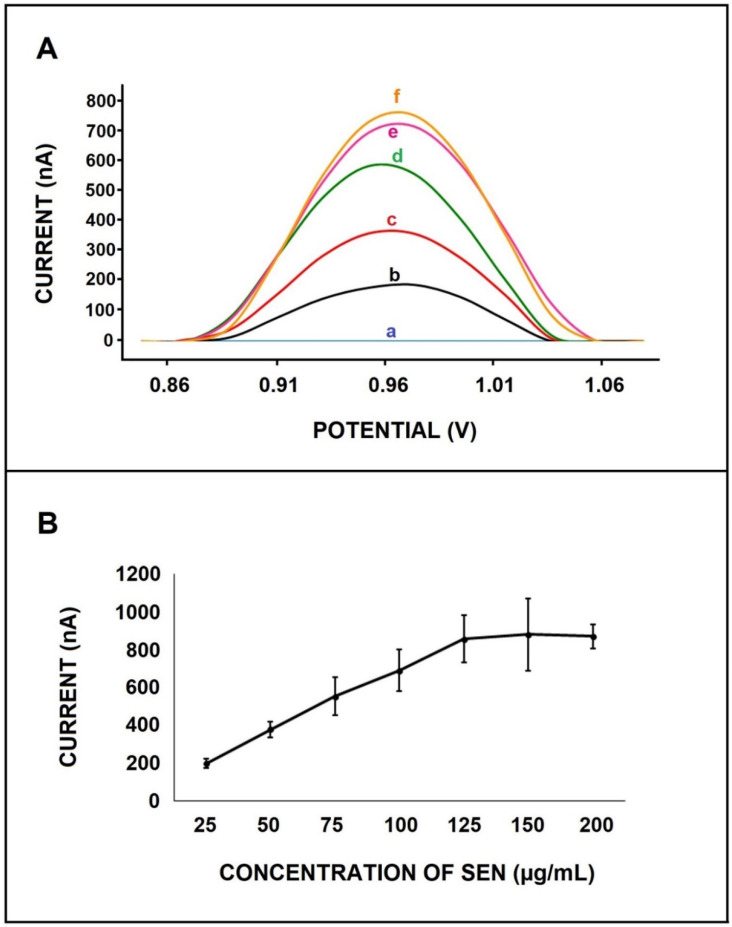
(**A**) Voltammograms representing the oxidation signal of SEN in the concentration range of 25–125 µg/mL; (a) 0, (b) 25, (c) 50, (d) 75, (e) 100, (f) 125 µg/mL SEN. (**B**) Line graph representing the average oxidation signal of SEN (*n* = 3) in the concentration range of SEN from 25 to 200 µg/mL.

The effect of SEN concentration upon electrode response was investigated. PGEs were immersed into the SEN solution in varying concentrations from 25 to 125 µg/mL (shown in Figure S3). The LOD was calculated in compliance with Miller and Miller [26] with a regression equation y = 5.73x + 0.46 (R^2^ = 0.9963) and found to be 7.19 µg/mL. In order to examine the effect of the dissolution medium of SEN on the sensitivity with LOD, SEN was dissolved in methanol, as reported in earlier studies [27,28,29,30,31]. The diluted solutions of SEN in varying concentrations from 25 to 125 µg/mL were prepared in PBS, and the SEN signal at each concentration was measured (shown in Figure 3). The LOD was calculated as 5.45 µg/mL, with a regression equation y = 6.51x + 46.21 (R^2^ = 0.9979).

**Figure 4:**
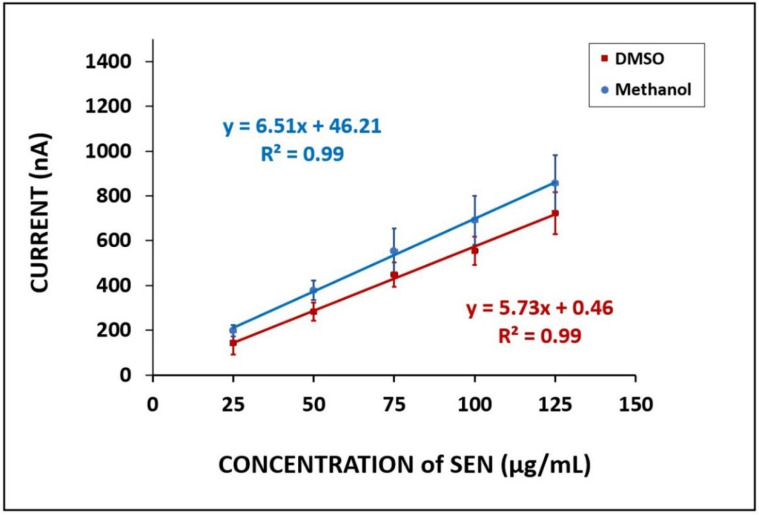
The calibration plots representing the average oxidation signal of SEN dissolved in different media: DMSO and methanol. SEN concentrations varied from 25 to 125 µg/mL.

Regarding these data, higher oxidation signals and lower LOD with higher sensitivity was obtained when SEN was dissolved in methanol in contrast with DMSO (Figure 4, Table S1). The sensitivity value was calculated and was found to be 5.73 µA·mL/µg and 6.51 µA·mL/µg with SEN dissolved in DMSO and methanol, respectively. Hence, the stock solution of SEN was prepared in methanol for our further experiments.

**Figure 5:**
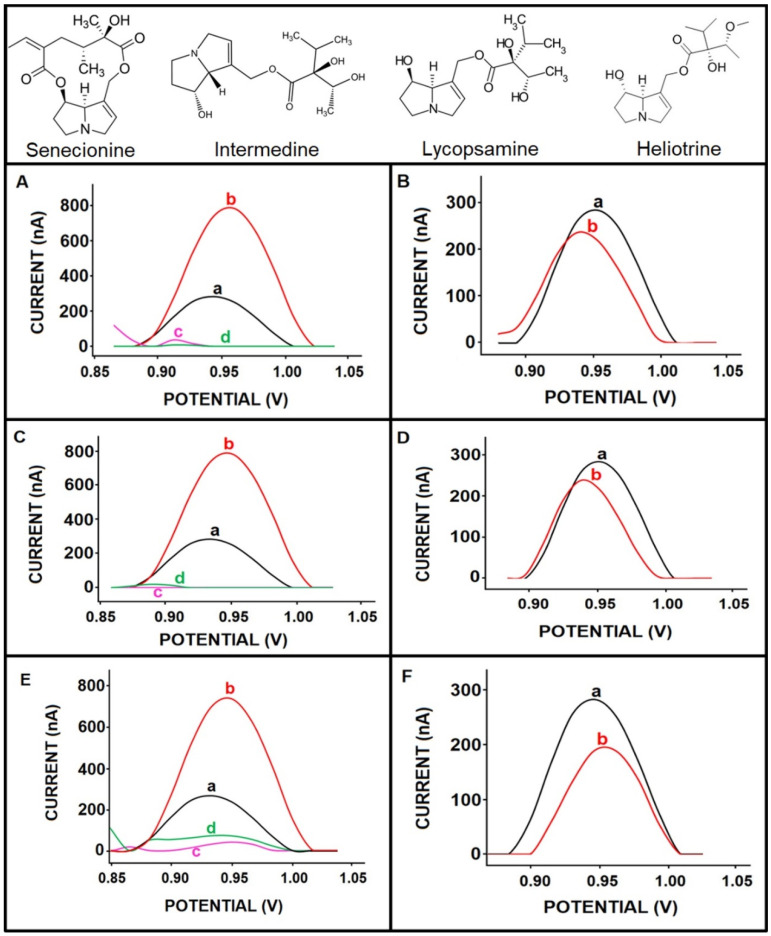
Voltammograms related to the selectivity of our assay in the presence of potential interferents individually (**A**,**C**,**E**) or in mixture samples (**B**,**D**,**F**) with the chemical structure of SEN and interferents. (**A**) The oxidation signal of (a) 25 µg/mL SEN, (b) 100 µg/mL SEN, (c) 25 µg/mL intermedine, (d) 100 µg/mL intermedine. (**B**) The oxidation signal of 25 µg/mL SEN (a) in the absence of intermedine and (b) in the presence of mixture sample containing 25 µg/mL SEN and 25 µg/mL intermedine. (**C**) The oxidation signal of (a) 25 µg/mL SEN, (b) 100 µg/mL SEN, (c) 25 µg/mL lycopsamine, (d) 100 µg/mL lycopsamine. (**D**) The oxidation signal of 25 µg/mL SEN (a) in the absence of lycopsamine and (b) in the presence of mixture sample containing 25 µg/mL SEN and 25 µg/mL lycopsamine. (**E**) The oxidation signal of (a) 25 µg/mL SEN, (b) 100 µg/mL SEN, (c) 25 µg/mL heliotrine, (d) 100 µg/mL heliotrine. (**F**) The oxidation signal of 25 µg/mL SEN (a) in the absence of heliotrine and (b) in the presence of mixture sample containing 25 µg/mL SEN and 25 µg/mL heliotrine.

The selectivity of our electrochemical assay to SEN over to other pyrrolizidine alkaloids, such as intermedine, lycopsamine, and heliotrine, was studied individually as well as in mixture samples containing 25 µg/mL of SEN and 25 µg/mL of intermedine, lycopsamine, or heliotrine. The resulting voltammograms are shown in Figure 5. The oxidation signals of 25 µg/mL and 100 µg/mL SEN were measured as 209.67 ± 15.76 nA (RSD%, 7.52%, *n* = 6) and 718.75 ± 48.30 nA (RSD%, 6.72%, *n* = 6), respectively. In addition, the oxidation signals of 25 and 100 µg/mL intermedine (Figure 5A(c,d)), lycopsamine (Figure 5C(c,d)), and heliotrine (Figure 5E(c,d)) were also measured individually in buffer medium. No oxidation signal of intermedine (Figure 5A(c,d)) and lycopsamine (Figure 5C(c,d)) was measured in the same voltammetric scale from +0.85 to 1.0 V at both a concentration value of 25 µg/mL and 100 µg/mL. However, there were very negligibly low signals measured (i.e., 7.33 ± 17 nA and 61.50 ± 76.53 nA) in the presence of 25 and 100 µg/mL heliotrine alone (Figure 5E(c,d)); these signals were not found to be significant enough to be evaluated. The lack of interferents signal in this potential range has been a substantial datum in terms of the selectivity of our electrochemical assay. Moreover, the selectivity was tested in the mixture of SEN/intermedine (1:1), SEN/lycopsamine (1:1), and SEN/heliotrine (1:1) under the same conditions, and the oxidation signals were examined. The oxidation signal of SEN in the absence/presence of these pyrrolizidine alkaloids are listed in Table 1.

There were negligible changes in the response of the electrode in the mixture samples containing SEN and other PAs, respectively lycopsamine, intermedine, and heliotrine. The value of difference, which was calculated according to Equation (1), is also shown in Table 1. A small decrease in the SEN oxidation signal was recorded in the presence of each interferent that was under 5%. Hence, the interferent effect could be accepted as being negligible according to these data. It was concluded that this electrochemical assay could provide a selective protocol for SEN detection even in the presence of other pyrrolizidine alkaloids that have similar chemical structures to SEN (see Figure 5).

(1) $$Difference=I_{oxidation\;signal\;of\;SEN}-I_{oxidation\;signal\;of\;SEN\;in\;the\;presence\;of\;interferent}$$

## 4. Voltammetric Detection of SEN in Flour and Herbal Tea Samples

Our electrochemical assay in combination with a single-use electrode was applied to detect SEN in flour sample. The standard addition method was then applied by the addition of the required amount of SEN standard solution into the flour sample. Accordingly, the oxidation signal of SEN was measured under optimum conditions, and voltammograms are given in Figure S4. The control experiments were carried out in 0.5% flour sample in the absence of SEN (Figure S4a). Since a background signal was obtained in the control sample, which overlapped with the SEN signal at almost the same peak potential (i.e., +0.97 V), the alteration at current value was calculated for each SEN concentration according to the Equation (2).

(2) $$∆I=I_{sample\;with\;SEN\;}-I_{control\;sample\;without\;SEN}$$

**Figure 6:**
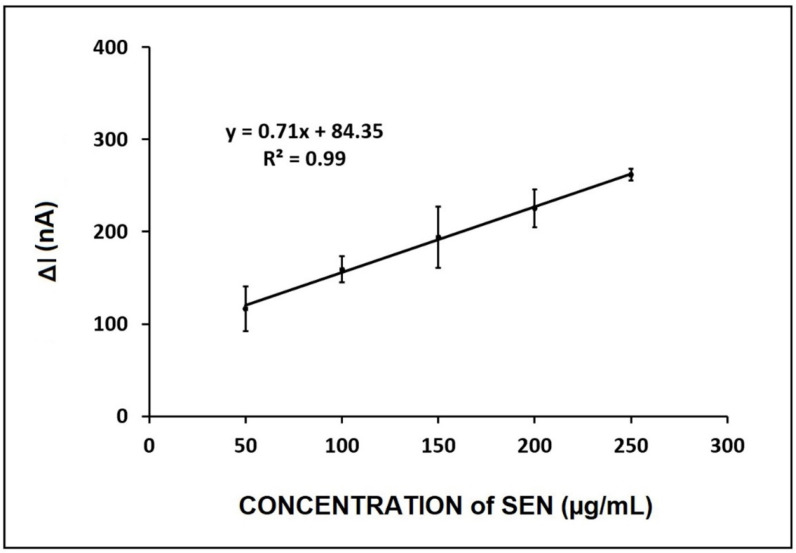
The calibration plot representing the average oxidation signal (*n* = 3) in the concentration range of 50–250 µg/mL SEN prepared in 0.5% flour sample.

Based on the calibration plot (Figure 6), the LOD was calculated following the method of Miller and Miller [26], with a regression equation y = 0.71x + 84.35 (R^2^ = 0.9973). The LOD was found to be 12.28 µg/mL.

**Figure 7:**
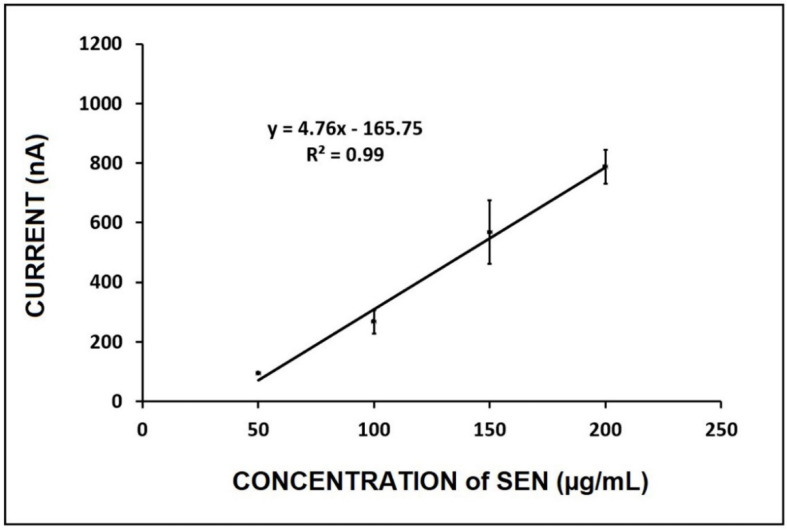
The calibration plot representing the average oxidation signal (*n* = 3) in the concentration range of 50–200 µg/mL SEN in 1:100 diluted linden tea sample.

Next, our method was applied for the detection of SEN in the linden tea sample. Similarly, the standard addition method was applied by the addition of the required amount of SEN standard solution into 1:100 diluted linden tea sample, and the SEN oxidation signal was measured under optimum conditions and voltammograms are given in Figure S5. There was no background signal in the absence of SEN; thus, measured current was used directly to obtain a calibration plot (Figure 7). LOD was calculated using the equation of y = 4.76x−165.75 (R^2^ = 0.9905) and was found to be 18.98 µg/mL. 

## 5. Conclusions

To the best of our knowledge, this study is the first electrochemical assay developed for the detection of SEN. The electrochemical detection of senecionine was also performed in food products for the first time in the literature. Senecionine detection was carried out by using the differential pulse voltammetry (DPV) technique with a pencil graphite electrode (PGE). The selectivity of our electrochemical assay to SEN over other pyrrolizidine alkaloids, such as intermedine, lycopsamine, and heliotrine, was tested, and it was concluded that our electrochemical assay could be used for SEN detection in the presence of intermedine, lycopsamine, and heliotrine, which have similar chemical structures to SEN.

In our study, the sensitivity value was calculated and found to be 6.51 µA·mL/µg with SEN dissolved in methanol. The electrochemical detection of SEN was presented in a high sensitivity with a good selectivity, resulting in very reproducible results (e.g., the RSD% value as 7.52% (*n* = 6) was recorded in the sample containing 25 µg/mL SEN).

In the study, the analysis was carried out without applying any surface modification, a long period of time, or a multi-step pretreatment process. SEN analysis in foods has been performed by laborious methods such as ELISA, liquid chromatography tandem mass spectroscopy, HPLC-MS/MS, reversed-phase high-performance liquid chromatography (RP-HPLC), and micellar electrokinetic capillary chromatography (MEKC). Some earlier studies related to the detection of alkaloids is presented in Table 2. In comparison with earlier reports on SEN detection protocols [27,28,29,30,31,32,33,34], rapid and sensitive voltammetric detection of SEN was performed in the present study by an easy-to-use and low-cost disposable sensor in combination with an electrochemical measurement protocol. Our electrochemical assay was also successfully applied for SEN detection in food products, such as, flour and herbal tea. In comparison with the chromatographic methods in the literature, electrochemical methods have some advantages over these laborious methods, such as ELISA, mass spectroscopy, and HPLC-MS/MS. In particular, chromatographic methods have disadvantages, such as being expensive, using complex devices, taking a long time for analysis, and needing trained personnel. It is not yet possible to use chromatographic devices in on-site analysis. However, electrochemical methods have many advantages, such as being rapid (whole analysis results in only 30–40 min) and sensitive, relatively cheap devices, easy-to-use, and enabling on-site analysis.

Our collaborative research focused on the development of an electrochemical sensor and its implementation in chromatographic methods. Hence, it can be easily adapted to the determination of SEN and other pyrrolizidine alkaloids and will enable on-site analysis in the future. Further research could also be conducted on the detection of pyrrolizidine alkaloids with different types of electrochemical sensors by the incorporation of nanomaterials. It is possible to increase sensitivity towards the detection of pyrrolizidine alkaloids as well as to obtain a superior overall performance by the incorporation of different nanoparticles or other electrode surface modifiers, which could be the subject of our further study. As a conclusion, our electrochemical assay has a commercialization potential for the development of novel sensor technologies for screening different analytes in food products that are vital to food safety and human health.

## Figures and Tables

**Table 1:** The average oxidation signal of SEN in the absence/presence of intermedine, lycopsamine, or heliotrine with RSD% (*n* = 6) and values of difference at SEN signal.

Sample Content	Current (nA)	RSD%	Difference
25 µg/mL SEN	209.67 ± 15.76	7.52	-
25 µg/mL SEN in the presence of 25 µg/mL intermedine	202.67 ± 14.57	7.19	7 nA (3.3%)
25 µg/mL SEN in the presence of 25 µg/mL lycopsamine	200.00 ± 9.17	4.58	9.67 nA (4.6%)
25 µg/mL SEN in the presence of 25 µg/mL heliotrine	208.50 ± 20.24	9.71	1.17 nA (0.5%)

**Table 2:** The comparison of our assay over some earlier reports on SEN detection protocols with LOD and application to real sample.

Method	Alkaloid	LOD	Application to Real Sample	Reference
Liquid chromatography-ion trap mass spectroscopy	Senecionine	0.0237 µg/mL	Honey	[27]
HPLC-MS/MS	Senecionine and Senecionine N-Oxide	Senecionine: 57 ng/kg Senecionine N-Oxide: 59 ng/kg	Honey	[28]
Liquid chromatography tandem mass spectroscopy	Senecionine	1.32 µg/g	Medicinal plants	[29]
MEKC	Senecionine	1 µg/mL	Medicinal plant	[30]
RP-HPLC	Senecionine and Senecionine N-Oxide	Senecionine: 0.21 µg/mL Senecionine N-Oxide: 0.52 µg/mL	Medicinal plant	[31]
ELISA	Senecionine	68.3 fmol	Medical plants	[32]
ELISA	Senecionine	Parent PA: 31,200 ng/mL, Digested PA: 1190 ng/mL	Plants	[33]
HPLC-MS	Senecionine	0.002 mg/kg	Honey	[34]
DPV	Senecionine	5.45 µg/mL in buffer, 12.28 µg/mL in flour sample, and 18.98 µg/mL in linden tea sample	Flour and herbal tea	This study

## Data Availability

The data presented in this study are available within the article. Other data that support the findings of this study are available upon request from the corresponding author as well as co-authors.

## Supplementary Materials

The following are available online at https://www.mdpi.com/article/10.3390/mi12121585/s1, Figure S1: CVs recorded by PGE in (a) PBS in the absence of SEN, (b) 10 µg/mL SEN solution. The inset shows a blow-up of the oxidation signal of SEN, Figure S2: Voltammograms representing (a) the control experiment performed by PGE in PBS in the absence of SEN, the oxidation signal of 50 µg/mL SEN after immobilization onto the PGE surface during (b) 15 min, (c) 30 min, (d) 60 min, Figure S3: (A) Voltammograms representing the oxidation signal of SEN (its stock solution was prepared in DMSO) in the concentration range of 25–125 µg/mL; (a) 0, (b) 25, (c) 50, (d) 75, (e) 100, (f) 125 µg/mL SEN. (B) Line graph representing the average oxidation signal of SEN (*n* = 3) in the concentration range of SEN from 25 to 200 µg/mL, Table S1: Oxidation signals obtained from SEN in the range of 0–125 µg/mL prepared in different medium (*n* = 3), Figure S4: Voltammograms representing the oxidation signal of SEN prepared in 0.5% flour sample (a) in the absence of SEN (control experiment) and in the presence of (b) 50 µg/mL, (c) 100 µg/mL, (d) 150 µg/mL, (e) 200 µg/mL, (f) 250 µg/mL SEN in 0.5% flour sample, Figure S5: Voltammograms representing the oxidation signal of SEN prepared in 1:100 diluted linden tea sample (a) in the absence of SEN (control experiment) and in the presence of (b) 50 µg/mL, (c) 100 µg/mL, (d) 150 µg/mL, (e) 200 µg/mL SEN in 1:100 diluted linden tea sample.
