# Supplementary material for: Detection of Senecionine in Dietary Sources by Single-Use Electrochemical Sensor
Source: Micromachines (Basel). 2021 Dec 20;12(12):1585. doi: 10.3390/mi12121585 (PMC8709324; doi:10.3390/mi12121585)
Supplement: Supplementary file 1 [file micromachines-12-01585-s001.zip › micromachines-1448160-SM.pdf]

Supplementary Materials

Detection of Senecionine in Dietary Sources by Single-Use Electrochemical Sensor

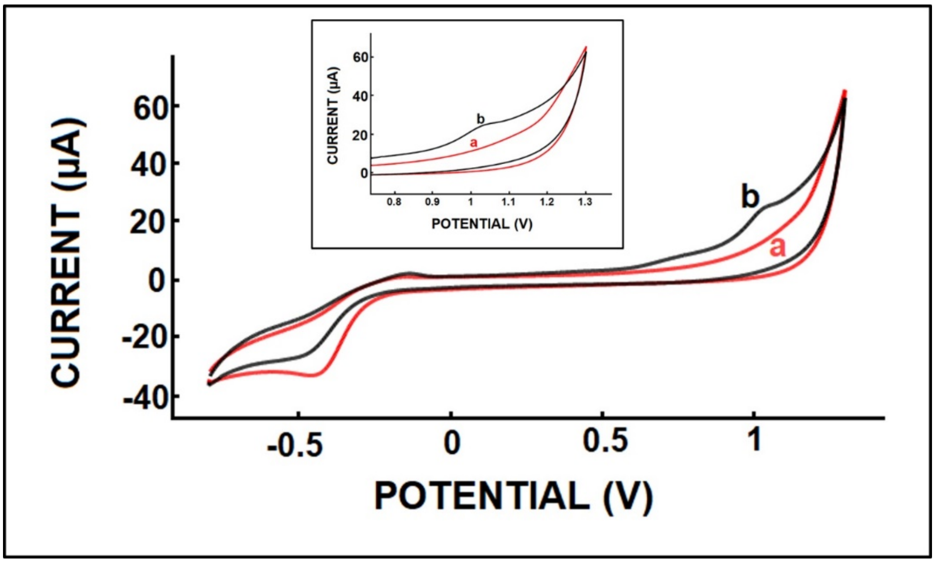

Fig S1. CVs recorded by PGE in (a) PBS in the absence of SEN, (b) 10 µg/mL SEN solution. The inset shows a blow-up of the oxidation signal of SEN.

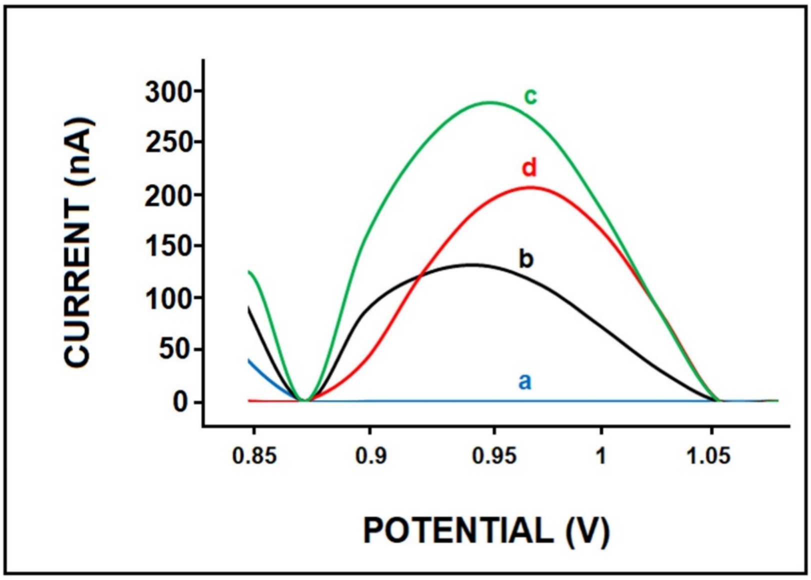

Figure S2. Voltammograms representing (a) the control experiment performed by PGE in PBS in the absence of SEN, the oxidation signal of 50 µg/mL SEN after immobilization onto the PGE surface during (b) 15 min, (c) 30 min, (d) 60 min.

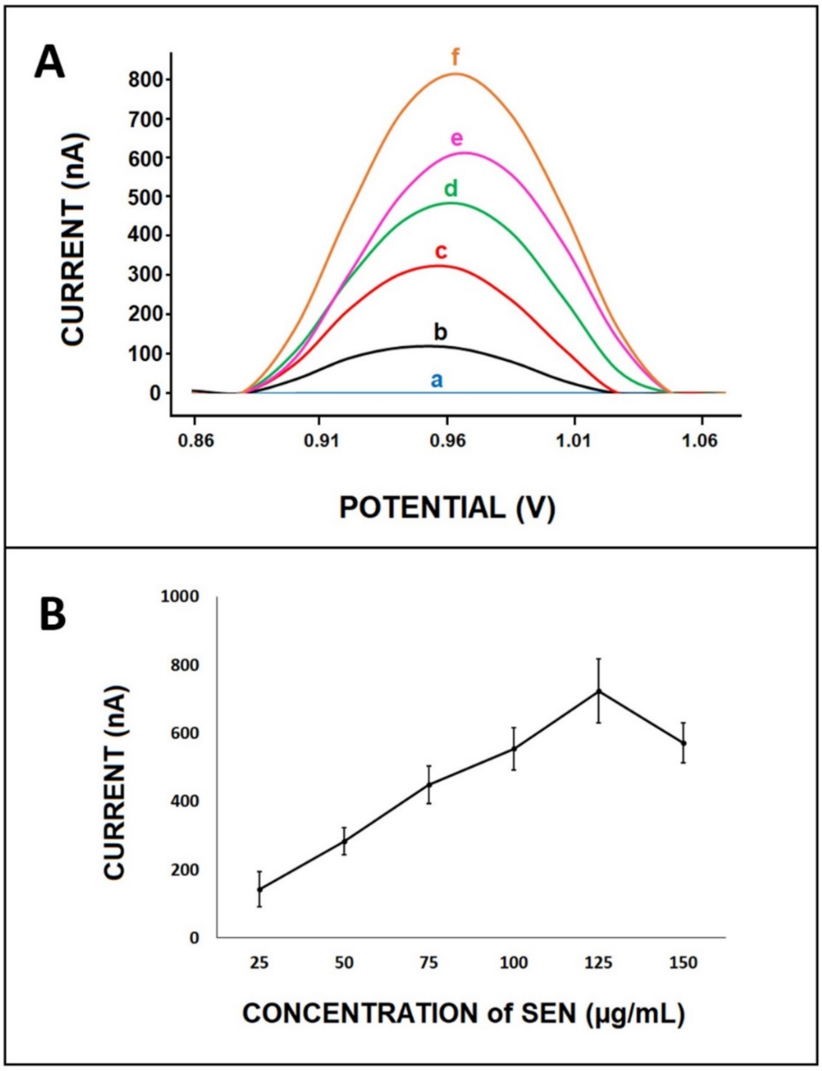

Figure S3. (A) Voltammograms representing the oxidation signal of SEN (its stock solution was prepared in DMSO) in the concentration range of 25–125 µg/mL; (a) 0, (b) 25, (c) 50, (d) 75, (e) 100, (f) 125 µg/mL SEN. (B) Line graph representing the average oxidation signal of SEN (n=3) in the concentration range of SEN from 25 to 200 µg/mL.

Table S1. The average oxidation signals of SEN (n=3) measured in the range of 0 – 125 µg/mL prepared in different medium.

Concentration of SEN (µg/mL)	Stock Solution in DMSO	Stock Solution in Methanol
	Current (nA)	Current (nA)
**25**	142.67 ± 52.31	197.80 ± 24.64
**50**	282.80 ± 40.20	377.67 ± 43.15
**75**	448.67 ± 54.50	553.00 ± 102.24
**100**	554.67 ± 62.01	690.60 ± 107.97
**125**	723.33 ± 94.08	856.00 ± 124.93

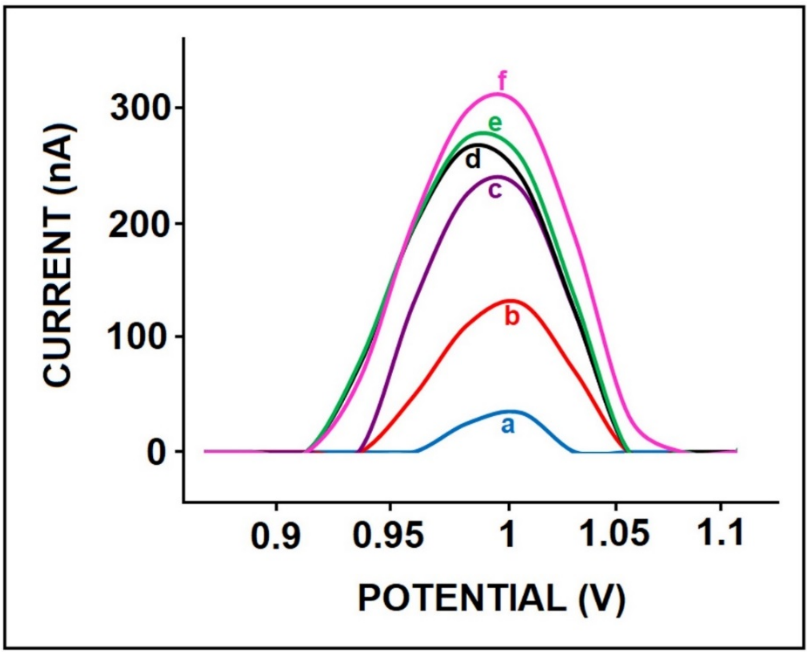

Fig S4. Voltammograms representing the oxidation signal of SEN prepared in 0.5 % sample of flour (a) in the absence of SEN (control experiment) and in the presence of (b) 50 µg/mL, (c) 100 µg/mL, (d) 150 µg/mL, (e) 200 µg/mL, (f) 250 µg/mL SEN in 0.5 % flour sample.

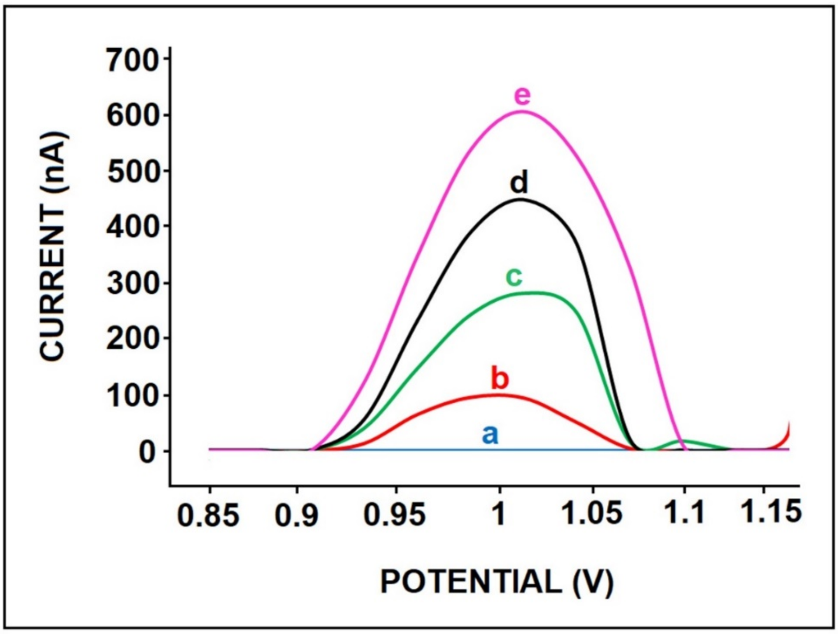

Fig S5. Voltammograms representing the oxidation signal of SEN prepared in 1:100 diluted sample of linden tea (a) in the absence of SEN (control experiment) and in the presence of (b) 50 µg/mL, (c) 100 µg/mL, (d) 150 µg/mL, (e) 200 µg/mL SEN in 1:100 diluted sample of linden tea.
